# Effect of Bitter Melon Extracts on Lipid Levels in Japanese Subjects: A Randomized Controlled Study

**DOI:** 10.1155/2018/4915784

**Published:** 2018-11-08

**Authors:** Hiroki Kinoshita, Yasuyuki Ogata

**Affiliations:** ^1^Department of Public Health, Graduate School of Medicine, The University of Tokyo, 7-3-1 Hongo, Bunkyo-ku, Tokyo 1138654, Japan; ^2^Imagine Global Care Corporation, 3-16-12 8th Fl. Roppongi Minato-ku, Tokyo 1060032, Japan

## Abstract

Dyslipidemia is exemplified by high levels of low-density lipoprotein cholesterol (LDL-C) and represents a risk factor for cardiovascular diseases and requires therapeutic intervention. Several experimental studies suggest that bitter melon (*Momordica charantia*) improves lipid metabolism in animal models of dyslipidemia and diabetes. This study evaluated the effects of bitter melon extracts on lipid metabolism following a 30-day treatment period in Japanese adults. This randomized, double-blind, placebo-controlled trial included 43 adult volunteers who received either 100 mg of hot-water extracts of bitter melon (*n *= 23) or a placebo (*n *= 20) three times daily for 30 days. The body weight, blood pressure, and levels of LDL-C and other blood parameters of each subject were measured before and after the study period. The results showed that the intervention group exhibited significantly lower LDL-C levels (*P *= 0.02) as compared with the control group, and there were no significant changes in either group in terms of body weight, body mass index, systolic pressure, diastolic pressure, total cholesterol, high-density lipoprotein cholesterol, triglycerides, or blood glucose. These results suggested that bitter melon extracts might effectively lower LDL-C levels in humans and exhibit potential therapeutic value for the management of dyslipidemic conditions.

## 1. Introduction

Cardiovascular diseases (CVDs) remain the leading global cause of death, with the World Health Organization projecting that 8,760,000 people died due to ischemic heart disease in 2015 [1]. Various studies demonstrated that dyslipidemia is a risk factor for CVD [2], with low-density lipoprotein cholesterol (LDL-C) having a greater effect than high-density lipoprotein cholesterol (HDL-C) or triglycerides (TGs). The US National Cholesterol Education Program Adult Treatment Panel III guidelines found that high LDL-C levels constitute a major risk factor for coronary heart disease and require clinical intervention [3].

Lipid-lowering drugs, such as statins, are used along with lifestyle interventions to treat high LDL-C levels, although the absence of clear symptoms is associated with poor drug adherence [4]. Furthermore, many patients have dyslipidemia and borderline dyslipidemia, which results in large numbers of patients with inadequately managed high LDL-C levels. Improvements in diet and exercise can be enhanced by avoiding saturated fats and cholesterol, as well as consuming greater proportions of plant stanols/sterols and soluble fiber [3]. Moreover, lower LDL-C levels can be achieved by consuming greater proportions of oats, avocados, nuts, soybeans, tomatoes, apples, and prunes [5–11].

Several studies report that bitter melon (*Momordica charantia*) can improve blood glucose levels and lipid metabolism in animal models of dyslipidemia and diabetes [12, 13]. Bitter melon belongs to the* Cucurbitaceae *family and is commonly eaten as a vegetable in Asia, Africa, and Latin America. Additionally, it has been used as a traditional herbal medicine for treating diabetes in India and China for ages [12]. Studies using mouse models suggest that bitter melon improves glucose and lipid metabolism by activating the translocation of GLUT4 to cell membranes in mouse L6 myotubes, 3T3-L1 adipocytes, skeletal muscle tissue, and the liver, as well as promoting AMP-activated protein kinase (AMPK) function [13–15]. Moreover, bitter melon reduces the mRNA levels of 11*β*-hydroxysteroid dehydrogenase type 1 (11*β*-HSD1) in the mouse liver, which reduces its excessive glucocorticoid activity and involvement in the development of obesity and insulin resistance [15]. Furthermore, patients with recently diagnosed diabetes and who consumed bitter melon in herbal supplements exhibited decreased plasma glycated hemoglobin levels and improved TG levels (vs. baseline) along with a modest effect on hypoglycemia [16]. Therefore, the authors of that report concluded that bitter melon helps ameliorate the diabetes-associated risk of CVD.

Several research groups evaluated the lipid-lowering effect of bitter melon in mouse and rat models of obesity and diabetes, providing evidence that its use can improve dyslipidemia (e.g., levels of TGs and LDL-C) and hyperglycemia [17, 18]. Additionally, bitter melon extract suppresses SREBP1c [18], which is thought to play an important role in the expression of lipid- constituting enzymes, synthesis of fatty acids, and accumulation of TGs. Furthermore, pretreatment of rats with a bitter melon polysaccharide extract reduced the size of isoproterenol-induced myocardial infarction, as well as serum levels of total cholesterol, TGs, and LDL-C. However, this treatment increased the activity of superoxide dismutase and catalase along with concomitant increases in proinflammatory cytokines and decreases in inflammatory markers, such as nitric oxide [12, 19]. These observations suggest that bitter melon might have a myocardial-protective effect, although no human study has examined the effects of bitter melon on human lipid metabolism. Therefore, the present randomized controlled study examined the effect of bitter melon extract on lipid metabolism in a group of Japanese adults.

## 2. Materials and Methods

### 2.1. Materials

This study used capsules containing bitter melon extract from Okinawa prefecture or placebo. The bitter melons were subjected to hot-water extraction and filtration along with the addition of starch hydrolysate as an excipient before being autoclaved and dried. Based on our research, the constituent (derived from bitter gourd) extracted with this method is considered a type of pectin (a plant cell-wall constituent). Because the extract has a unique appearance and taste, the study dose (100 mg of extract, approximately equivalent to 3 g of melon) was placed in white capsules that also contained microcrystalline cellulose, calcium stearate, and fine silicon dioxide as fillers. The placebo capsules were filled with starch hydrolysate.

### 2.2. Subjects

For this randomized controlled trial, 47 healthy Japanese adults (19 men and 28 women) were recruited according to the method described by Ursoniu et al. [20]. Subject recruitment was coordinated by Huma Corporation (Minato Ward, Tokyo, Japan). The inclusion criteria were (1) age between 40 and 74 years and (2) willingness to provide written informed consent to participate after receiving a sufficient explanation regarding the purpose and procedures of the study. The exclusion criteria were (1) receiving continuous drug therapy (e.g., pranlukast hydrate, metformin, and lipid-lowering drugs), (2) exhibiting an allergic response to the study materials, (3) consuming supplements that might affect the study parameters (based on the discretion of the attending physician), (4) having had digestive organs surgically removed, (5) having had the presence or possibility of pregnancy and/or breast feeding, and (6) having participated in another clinical study within the previous 3 months.

### 2.3. Study Design and Parameters

The study protocol was approved by the Ethics committee of Imagine Global Care Corporation and was pre-registered in the University Hospital Medical Information Network Clinical Trials Registry (UMIN000026636). All participants provided written informed consent, and the study was performed in accordance with the 2013 Declaration of Helsinki.

The subjects were randomly assigned. This was specifically done by placing participants in either the control group or the intervention group using a computer-generated randomized number table. The subjects, study doctor, and data analyzer were blinded to subject assignments. Starting on day 1, the subjects consumed three capsules daily (100 mg) for 30 days, with each capsule containing either bitter melon extract or placebo. Blood testing and measurements of weight and blood pressure were performed on days 1 (the first visit) and 30 (the second visit).

Subjects were instructed to not consume sweet food or drinks after 22:00 on the night before blood testing. The values of total cholesterol, HDL-C, LDL-C, TGs, glucose, and glycated hemoglobin were also determined. All biochemical tests were conducted by LSI Medience Corporation (Tokyo, Japan) using Stacia, an automated clinical testing machine.

### 2.4. Statistical Analysis

Pearson's chi-square test was used to compare the male: female ratios between the control and intervention groups. An unpaired* t *test was used to compare baseline and post intervention body weight, blood pressure, and biochemical parameters between the control and intervention groups as well as the changes in these values for each group. Confounding effects were evaluated using multiple regression analysis (forced entry method), with the change in LDL-C levels used as the dependent variable, and sex, age, and baseline body mass index used as independent variables.

The Japan Atherosclerosis Society defines hypercholesterolemia as ≥140 mg/dL LDL-C and borderline hypercholesterolemia as 120–139 mg/dL LDL-C. Therefore, we additionally analyzed subjects whose baseline LDL-C was equal to or exceeded 120 mg/dL and determined the difference in changes in LDL-C between the control and intervention subjects. Differences were considered statistically significant at* P *< 0.05, and all analyses were performed using SPSS software (v17.0; SPSS, Inc., Chicago, IL, USA).

## 3. Results

**Figure 1:**
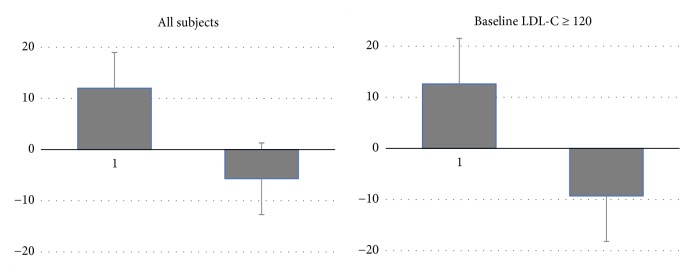
**Changes in LDL‐C**. p=0.02 for both all subjects and those with baseline LDC‐C ≥ 120.

Among the 47 recruited subjects, two were excluded because they were receiving continuous drug therapy (pranlukast hydrate and metformin), and two other were excluded after failing to attend the second visit. There was no significant difference in the male: female ratios of the control (*n *= 20) and intervention (*n *= 23) groups (Table 1).  Table 2 shows the changes in metabolic parameters of the subjects from baseline to post intervention. No significant differences were observed between the control and intervention groups at baseline. The intervention group showed significantly decreased LDL-C levels at the second visit (-5.7 ± 18.5 mg/dL) as compared with the control group (+12 ± 27.3,* P *= 0.02). There was no significant difference in changes in body weight, BMI, blood pressure, total cholesterol, HDL-C, TGs, glucose, and glycated hemoglobin between the control and intervention groups. Multiple regression analysis revealed that the intervention group had a significantly increased likelihood of lower LDL-C levels after adjusting for sex, age, and baseline body mass index (Table 3). Baseline LDL-C ≥140 mg/dL was observed for 6 subjects in the control group and 9 subjects in the intervention group compared with 7 subjects in each group at the second visit. Furthermore, baseline LDL-C ≥120 mg/dL was observed for 10 subjects in the control group and 14 subjects in the intervention group compared with 11 subjects in each group at the second visit. Among the subjects with baseline LDL-C equal to or exceeding 120 mg/dL, the mean changes were +12.6 ± 25.8 mg/dL in the control group and -9.3 ± 17.9 mg/dL in the intervention group (*P *= 0.02).  Figure 1 shows the changes in LDL-C levels in all subjects in the control and intervention groups and those with baseline LDL-C ≥120 mg/dL.

## 4. Discussion

Bitter melon has been used in traditional Indian and Chinese medicine since ancient times for the treatment of various ailments, including gastrointestinal complaints, constipation, dermatitis, cough, and diabetes [21]. However, several clinical studies have failed to show clear pharmacological effects.

Our research revealed that the component extracted from bitter melon in this study was one form of pectin. It has long been known that soluble fiber, including pectin, effectively lowers LDL-C. Brown et al. conducted a meta-analysis of four types of soluble fiber (pectin, oat bran, guar gum, and psyllium) and found that all reduce LDL-C to the same extent [22]. Namely, intake of 3 g of soluble fiber per day reduced LDL-C by roughly 5 mg/dL. Though soluble fiber also lowers HDL- C, the decrease is extremely slight, and there is no effect on TGs. This study was consistent with the results of previous research on soluble fiber, demonstrating that although bitter melon extract reduced LDL-C, there was no effect on HDL-C or TGs. The daily dose of bitter melon extract used in the present study was 300 mg—a small amount compared with that in most previous studies, in which quantities were in grams—suggesting that soluble fiber from bitter melon extract is effective at reducing LDL-C even at a lower dose.

There are several possible mechanisms involved in the reduction in HDL-C by soluble fiber. First, the theory that fiber promotes bile acid excretion, thereby reducing cholesterol, has been advocated for years [23]. The idea is that because blood cholesterol is used in the synthesis of bile acid, highly viscous soluble fiber adheres to and helps excrete bile acid enveloping cholesterol. However, some scholars argue that the amount of bile acid excretion is insufficient to explain the reduction in cholesterol [24]. It was further reported that soluble fiber increases the number of apo B/E receptors, which bind to LDL-C and accelerate the LDL-C metabolic turnover rate [25, 26]. Effects from improved insulin sensitivity [27] and inhibition of cholesterol synthesis in the liver due to the formation of short-chain fatty acids through fermentation of fiber in the large intestine have also been indicated [28, 29]. Although we observed significant decrease in LDL-C levels in the present study, there was no change in blood glucose levels, suggesting that reduced LDL-C levels might not be mediated by insulin resistance.

Previous studies evaluated the effects of bitter melon on dyslipidemia using rats. Bitter melon treatment of diabetic rats normalized the increase in nonesterified cholesterol, TGs, LDL, and phospholipids [18, 30, 31]. Additionally, increased mitochondrial biogenesis could be a pathway associated with increased lipid metabolism and utilization, with several genes, including* PPARγ*, involved in the regulation of this process [32, 33]. Bitter melon treatment of rats increased the expression of PPAR*γ* coactivator (PGC1*α*) and fatty acid-binding protein 1, and in this context, PGC1 family members influence hepatic metabolism by stimulating mitochondrial biogenesis and respiration in several types of cells while also altering biological pathways involved in oxidative metabolism [33]. In rat-based studies, test animals that received a high-fat diet and bitter melon extract displayed reduced levels of plasma TGs, cholesterol, and free fatty acids [34]. These results suggest that bitter melon extract might improve dyslipidemia in humans.

This study had several limitations. First, the small sample size of the study suggests that caution is needed in generalizing the applicability of the study results. Using purified samples of bitter melon extract might improve the interest of volunteers in enrolling in follow-up studies. Second, the dose of 300 mg per day used in this study may be too low compared with that in previous studies, in which several grams were administered daily. In addition, the administered dose in this study was not adjusted for individual body weight and was unrelated to the dose used in tradomedical use of bitter melon. Previous studies used doses of ~150 mg/kg [12] and between 500 mg/kg and 2,000 mg/kg [16, 35].

Therefore, it is important to perform additional studies to determine the mechanisms through which bitter melon lowers LDL-C levels, as well as its effects on other indices related to the lipid profile.

## 5. Conclusion

The water-soluble extract of bitter melon significantly decreased LDL-C levels as compared with the control (placebo) group in humans. Therefore, bitter melon might be useful in reducing the risks of cholesterol-mediated diseases, including CVDs.

## Figures and Tables

**Table 1:** Number and age of male and female subjects in the control and intervention groups.

	Control group		Intervention group		Overall	
	No.	Age	No.	Age	No.	Age
Male	9	62.9 ± 6.0	10	60.4 ± 6.4	19	61.6 ± 6.2
Female	11	53.7 ± 6.5	13	49.8 ± 5.7	24	51.6 ± 6.3
Total	20	57.8 ± 7.7	23	54.4 ± 7.9	43	56.0 ± 7.9

**Table 2:** Change in metabolic parameters after intervention with bitter melon.

		Control group (n = 20)	Intervention group (n = 23)	p-value
Body weight	Baseline	66.9 ± 14.5	62.5 ± 13.7	0.3
	Post intervention	66.6 ± 14.1	62.1 ± 13.5	0.3
	Change	-0.3 ± 1.1	-0.4 ± 1.2	0.6
Body mass index, kg/m^2^	Baseline	24.0 ± 3.9	23.1 ± 4.6	0.5
	Post intervention	23.9 ± 3.7	22.9 ± 4.5	0.5
	Change	-0.1 ± 0.4	-0.2 ± 0.5	0.6
Systolic pressure, mmHg	Baseline	131.3 ± 20.2	131.3 ± 18.0	0.7
	Post intervention	130.7 ± 19.2	128.1 ± 22.7	0.7
	Change	-2.8 ± 14.7	-3.2 ± 14.6	0.9
Diastolic pressure, mmHg	Baseline	83.0 ± 15.3	81.2 ± 10.3	0.7
	Post intervention	83.3 ± 12.6	79.2 ± 11.3	0.3
	Change	0.3 ± 7.9	2.0 ± 8.7	0.4
Total cholesterol, mg/dL	Baseline	216.2 ± 34.5	224.3 ± 35.6	0.5
	Post intervention	220.8 ± 34.4	225.1 ± 38.8	0.7
	Change	4.6 ± 31.2	0.8 ± 19.1	0.6
LDL-C, mg/dL	Baseline	119.9 ± 28.7	129.0 ± 28.9	0.3
	Post intervention	131.9 ± 32.1	123.4 ± 32.0	0.4
	Change	12.0 ± 27.3	-5.7 ± 18.5	0.02
HDL-C, mg/dL	Baseline	68.3 ± 25.0	70.8 ± 19.4	0.7
	Post intervention	70.9 ± 20.2	69.6 ± 19.1	0.8
	Change	2.6 ± 15.6	-1.3 ± 8.4	0.3
Triglycerides, mg/dL	Baseline	116.5 ± 77.8	132.8 ± 108.4	0.3
	Post intervention	108.6 ± 61.6	192.1 ± 229.8	0.12
	Change	7.9 ±192.3	59.3 ± 200.4	0.17
Glucose, mg/dL	Baseline	111.8 ± 38.7	106.7 ± 38.4	0.7
	Post intervention	108.2 ± 34.3	108.2 ± 44.2	1.0
	Change	-3.6 ± 9.9	1.5 ± 12.3	0.15
Glycated hemoglobin, %	Baseline	5.9 ± 1.1	5.8 ± 1.1	0.8
	Post intervention	5.9 ± 1.0	5.8 ± 1.1	0.7
	Change	0 ± 0.1	0 ± 0.1	0.7

**Table 3:** Multiple linear regression analysis of factors related to changes in LDL-C.

	*B*	*SE*	*β*	*P*
Sex (male = 0; female = 1)	–10.75	9.56	–0.22	0.27
Age	–0.99	0.60	–0.32	0.11
Group (control = 0; intervention = 1)	–21.18	7.33	–0.44	**0.006**
Body mass index	–0.31	0.91	–0.05	0.74

R^2^ = 0.2. Bold values represent significant differences.

*β*: standardized regression coefficient; B: unstandardized regression coefficient; LDL-C: low-density lipoprotein cholesterol; SE: standard error.

## Data Availability

The data used to support the findings of this study are included within the article.
